# Speech emotion analysis using convolutional neural network (CNN) and gamma classifier-based error correcting output codes (ECOC)

**DOI:** 10.1038/s41598-023-47118-4

**Published:** 2023-11-21

**Authors:** Yunhao Zhao, Xiaoqing Shu

**Affiliations:** Department of Chinese Language & Literature, The Catholic University of Korea, 43 Jibong-Ro, Gyeonggi-Do, Bucheon-Si, 14662 Republic of Korea

**Keywords:** Computational science, Computer science, Information technology

## Abstract

Speech emotion analysis is one of the most basic requirements for the evolution of Artificial Intelligence (AI) in the field of human–machine interaction. Accurate emotion recognition in speech can be effective in applications such as online support, lie detection systems and customer feedback analysis. However, the existing techniques for this field have not yet met sufficient development. This paper presents a new method to improve the performance of emotion analysis in speech. The proposed method includes the following steps: pre-processing, feature description, feature extraction, and classification. The initial description of speech features in the proposed method is done by using the combination of spectro-temporal modulation (STM) and entropy features. Also, a Convolutional Neural Network (CNN) is utilized to reduce the dimensions of these features and extract the features of each signal. Finally, the combination of gamma classifier (GC) and Error-Correcting Output Codes (ECOC) is applied to classify features and extract emotions in speech. The performance of the proposed method has been evaluated using two datasets, Berlin and ShEMO. The results show that the proposed method can recognize speech emotions in the Berlin and ShEMO datasets with an average accuracy of 93.33 and 85.73%, respectively, which is at least 6.67% better than compared methods.

## Introduction

Speech is one of the most basic means of human interaction, which can implicitly convey the feelings of the speaker to the listener. With the expansion of AI techniques, human–machine interaction systems have also been developed, and these systems must be able to process speech for improving interaction^1^. By analyzing the emotions hidden in speech, the feedback of intelligent systems can be improved and the information obtained from it can be used to make the output of these systems understandable by people^2,3^. Sentiment extraction can be useful in areas such as online support, lie detector systems, customer feedback analysis, and the like^4^. For this reason, achieving an accurate emotion extraction system can benefit from various practical aspects. Nevertheless, a significant part of the research in the field of sentiment analysis has focused on text processing, and speech analysis has received less attention^5^. The reason for this can be the higher complexity of speech analysis systems and the significant impact of noise (or background sounds) on system performance.

Achieving an accurate system for recognizing speech emotions requires solving several challenges. The complexity of emotional states is one of the first challenges. Because an emotional state can include a combination of several basic emotional states, and for this reason, recognizing basic emotions in speech is of considerable importance^6^. On the other hand, the extraction of speech features should be performed efficiently so that the relationship between verbal patterns and different emotional states can be interpreted based on it. The relatively large number of emotional states is another existing challenge that causes most learning techniques to be unable to correctly classify the speech’s emotional features^7^. In this paper, a hybrid solution is presented to solve the main challenges in the problem of speech emotions analysis. The difference between the current research and previous similar works can be investigated from feature extraction and classification. This method uses modulation analysis and deep learning techniques to extract speech features and it presents a new classification model based on the combination of ECOC and GC to overcome the challenge of, a large number of target classes. With these explanations, the contribution of the current paper can be summarized in the following cases:

- The proposed method in this article uses the combination of STM and entropy features of the speech signal to describe the emotional features, and the dimensions of these features are reduced by using a CNN. The features extracted through this CNN contain the most relevant features with emotional states.
- In this paper, a new classification model based on the combination of ECOC and GC is presented, which can be effective in solving classification problems with a large number of target classes. This classification model includes several GCs that are trained based on the ECOC matrix.

Based on the current knowledge of the authors, these two methods have not been used in previous research and can be considered as new approaches to fill the existing research gap. The rest of the paper is organized as follows: In section "Research background", the research background is studied. In section "Research method" the description of the proposed method is provided, and in section "Results", the findings of the research are discussed. Finally, sections "Discussion" and "Conclusion" contain the discussions and summary of the research findings, respectively.

## Research background

In^8^, speech emotion recognition was done with Artificial Neural Network (ANN) and Support Vector Machine (SVM). Since the effect of feature dimension reduction has been carefully evaluated, the effect of dimension reduction on these two models was compared. These features from the CASIA Chines Emotional Corpus dataset were extracted. In^9^, the Deep Neural network (DNN) classification method was applied to a custom dataset to recognize speech emotions. Only the Mel Frequency Cepstral Coefficients (MFCC) feature was considered for testing in this research.

In^10^ by using the Multiple Linear Regression (MLR) classification method, and with the combination of MFCC and MS features, seven emotions anger, hatred, happiness, fear, surprise, sadness, and normal state were reported with a recognition accuracy of 82.41%.

In^11^, the MFCC feature (which is widely used to analyze any speech signal) was employed to recognize emotions in speech. This set of features, which compared to other features, performed well for speech emotion recognition systems, includes 39 coefficients. In this research, Long Short-Term Memory (LSTM) was applied to recognize emotions. A multifaceted self-care method was proposed in^12^, which implements five convolution layers and one attention layer using MFCC for emotion recognition. This method applies functional descriptors to the output of the MFCC signal to increase recognition accuracy by combining them with features based on parts. This model is trained for the IEMOCAP dataset and considers only four emotions: happy, sad, angry, and neutral.

In^13^, DNN and Hidden Markov Model (HMM) classification methods using two MFCC and Epoch-based features were compared for four emotional states: happy, sad, angry, and normal in the IEMOCAP dataset. In^14^, emotion recognition was performed on four datasets, including the ShEMO dataset. The features used in this research are MFCC, formant, and prosodic features (such as the lowest and highest pitch and other pitch features) as well as a combination of these three features. However, in this paper, only the recognition of two emotional states, neutral and anger, was tested.

In^15^, the aggressiveness of a person's speech and state through mobile phone voice was discussed. It examines the feelings of anger and fears that cause stress from the speech available in various datasets such as BERLIN EMO-DB and recognizes the stress from the person's speech and expression. In this method, 13 MFCC-based features, 7 functional features, and 4 low-level features are used to describe the features of each speech signal. Then, SVM was used to classify the features. In^16^, emotion recognition in the speech was performed on six datasets, including ShEMO, and it was shown that the classification method can be made sensitive and dependent on a small number of phonetic labels that are clustered by the K-Means method and other feature components can be ignored. These specific phonetic components are taken from the MFCC feature and recognize speech emotions with DNN and SVM classification methods. The advantages of this reduction in dimensions are in reducing calculations, execution time, and cost, but; It does not achieve high accuracy.

In addition to speech, sentiment analysis can be done using other types of data. For example, in^17^, the issue of emotion evaluation with interaction levels in blended learning is discussed. In^18^, EEG brain signals are used to detect emotions. The methods presented in^19^ and^20^ have analyzed sentiments in texts. Also, the method presented in^21^ discusses the topic of emotion recognition in long dialogues.

## Research method

In this section, after describing the specification of datasets used in the research, the details of the proposed method for emotion analysis in speech signal of these datasets is presented.

### Data acquisition

In this research, the speech signals available in ShEMO^22^ and Berlin Emotional^23^ datasets were used as input for experiments. In the following, the specifications of the mentioned datasets are described first, and then the obtained results are analyzed.

The data set used from the Berlin dataset is 535 speech signals in six emotional categories, and a subset including 150 speech signals from this set was used in the proposed method. The selected speech signals are without background and the speakers include both male and female genders. The emotional states in this dataset are 1- Anger (31 samples), 2- Hatred (25 samples), 3- Fear (29 samples), 4- Joy (28 samples), and 5- Sadness (37 samples). The average length of speech signals in this dataset is equal to 3.051 s. Also, all samples of this dataset were recorded through two speakers (one male and one female). On the other hand, the ShEMO dataset has 3000 speech signals, all of which were collected from radio shows. This dataset's total sample length is 3 h and 25 min. This dataset was gathered through 78 Persian speakers (male and female) and includes six basic emotions, which are: 1- Anger (1059 samples), 2- Fear (38 samples), 3—happiness (201 samples), 4- neutral state (1028 samples), 5- sadness (449 samples) and 6- surprise (225 samples). Due to the shortness of a significant number of samples in this dataset, all samples with a length of less than one second were ignored. Following this process, 874 samples were removed from the dataset, and as a result, the experiments conducted in this research were performed on 2126 samples from the ShEMO dataset. In the experiments, the cross-validation technique was used with 10 iterations, and in each iteration, 90 samples were used for training the learning models and the remaining 10% were used for testing.

### The proposed method for emotion analysis in speech

**Figure 1:**
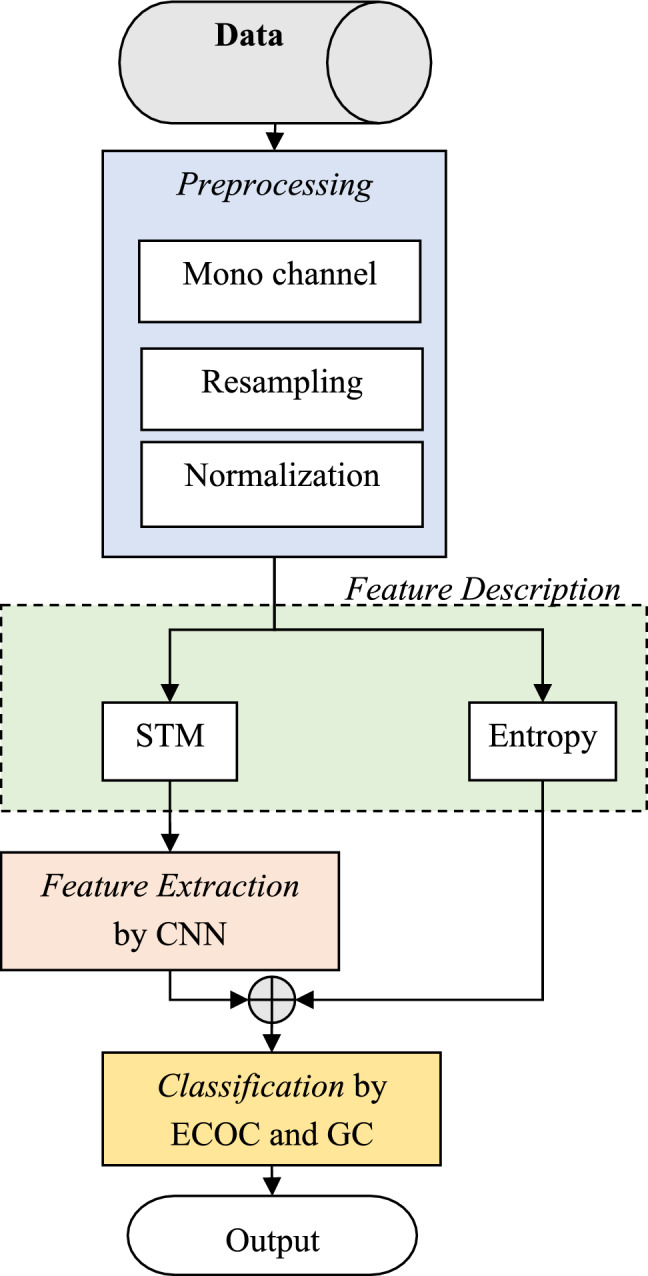
Flowchart of the proposed method.

A model for recognizing speech emotion can generally include pre-processing, feature extraction, and classification steps. Each of these processes is of great importance and the resulting combination should be able to efficiently extract emotional states with the highest possible accuracy. In the proposed method, the recognition of speech emotion is done using the following calculation steps (Fig. 1):

1. Preprocessing speech signal
2. Feature description based on spectro-temporal modulation and entropy features
3. Extracting features using CNN
4. Classification based on GC and ECOC

According to Fig. 1, the proposed method starts with preprocessing speech signals including converting to mono-channel, converting frequency by sampling, and signal normalization. In the second step, feature description is performed using two sets of features: entropy and spectro-temporal modulation. These two techniques process the normalized signal independently and extract features describing the signal. The features extracted through spectro-temporal modulation are reduced by using a CNN in the third step of the proposed method and are extracted as a vector containing significant emotional features in speech. This vector is merged with entropy features to recognize the emotion in the speech in the fourth step of the proposed method. For this purpose, a detection model based on the combination of ECOC and GC is used. This classification model includes a set of GCs that are trained based on the ECOC code matrix. In the rest of this section, the details of each step of the proposed method are described.

#### Preprocessing speech signal

The proposed method starts with preprocessing the input signals. This step includes three main steps:

- Converting to mono-channel
- Converting signal frequency
- Signal normalization

The purpose of the pre-processing step is to remove redundant data from the audio signal, convert all signals into a standard intermediate form and prepare them for use in the next steps. For this purpose, the processes of audio signal normalization, audio signal sampling, and transformation of two-channel signals into mono-channel vector form are used. In this way, at the beginning of the pre-processing step, the two-channel nature of the input audio signal is checked. If the signal is two (or more)-channel, we convert it to a mono-channel signal. Since audio signals are recorded in different conditions and by different devices; Therefore, the frequency of some input signals may be different from other signals. For this reason, in the second step of the pre-processing phase, all the vectors of the input signal are converted to the same frequency of 16 kHz. At the end of the pre-processing step, the signal vector is converted into a vector with zero mean and unit variance, so that the special conditions of a signal (high or low volume) are eliminated as much as possible.

#### Feature description based on spectro-temporal modulation and entropy features

In the second step, each speech signal is described with entropy features and spectro-temporal modulation. Each of these two techniques processes the pre-processed signal independently and extracts the descriptive features of the signal, which are described below.

##### Extraction of entropy features

The first feature set used to describe speech signal features is entropy features. In the proposed method, two types of features, approximate entropy, and sample entropy, are used to describe the general characteristics of each speech signal. Approximate entropy can describe speech signals based on the features of certainty, chaos, or randomness. A high approximate entropy value indicates disorder and a high level of chaos in the signal, while a low approximate entropy value indicates repetitive changes in the signal. On the other hand, sample entropy is a modified version of approximate entropy that can be used to evaluate the complexity of physiological and speech time series signals. Compared to approximate entropy, this criterion has two advantages: independence from the length of the data and relative implementation without problems. In^24^ calculation of these entropy values is explained; Therefore, it is omitted to deal with the details of the calculation of these features.

##### Feature description based on spectro-temporal modulation

The second set of features utilized to describe audio signal features is spectro-temporal modulation. This technique also performs the feature description process using auditory modeling and includes two basic processing steps:

- Modeling the human auditory system
- Generating temporal modulation based on the Auditory Spectrogram (AS)

In the first step, the human auditory system is modeled, during which the speech signal is converted into a neural pattern, i.e., AS.

AS is a time–frequency distribution along the tonotopic axis or logarithmic frequency, which is obtained by applying three stages of transformation on the input signal. In the second stage, the temporal modulation content is obtained through AS and by applying wavelet transformation to each line of the AS.

*Modeling the human auditory system*: The process of modeling the human auditory system consists of three main steps, which are based on the initial stages of sound processing by humans. In the first step, a constant Q transformation is applied to the input signal. This transformation is done using the filter bank, in all of the filters, the ratio of the central frequency and resolution is always a constant value. In the proposed method, 96 overlapped filters are used, whose central frequencies are linearly and uniformly distributed. To properly distribute these filters, the logarithmic frequency axis is divided into the following four octave ranges:

- Octave 1: 100 to 200 Hz
- Octave 2: 200 to 400 Hz
- Octave 3: 400 to 800 Hz
- Octave 4: 800 to 1600 Hz

All the 96 mentioned filters are distributed on the logarithmic frequency axis in such a way that they can cover these four octaves. If we consider $f$ as the logarithmic frequency of this filter bank, then the impulse response of each filter can be expressed as ${h}_{cochlea}\left(t,f\right)$. Considering the impulse response caused by each filter and s(t) as the input speech signal, the output of the cochlear filter can be calculated as follows^25^:

1 $$y_{{{\text{cochlea}}}} \left( {{\text{t}},\;{\text{f}}} \right) = {\text{s}}\left( {\text{t}} \right){*}_{{\text{t}}} {\text{h}}_{{{\text{cochlea}}}} \left( {{\text{t}},\;{\text{f}}} \right)$$

where *t represents a twist in the time domain. In this way, by calculating the output of the cochlear filter, the first stage of modeling the auditory system is completed. In the second step, the output obtained from the previous step (i.e., ${y}_{\mathrm{cochlea}}\left(\mathrm{t},\mathrm{f}\right)$) is converted into an auditory neural pattern by a hair cell. Using this process, the cochlear output can be modeled as an intracellular pattern. This transformation can be implemented through the following steps: First, the output obtained from each filter is derived with respect to high-pass $\left(t,f\right)$). This action works as a high-pass filter. Then, by applying a non-linear compress function such as ${gh}_{c}(.)$ on the output obtained from the previous step, ion channels can be modeled. The compress function ${gh}_{c}\left(.\right)$ is defined as follows^25^:

2 $$gh_{c } \left( f \right) = \frac{1}{{1 + e^{ - \gamma *f} }} - 0.5$$

Finally, by using a low-pass filter ${\mu h}_{c\left(t\right)}\left(0\right),$ the output of hair cells in the auditory system can be modeled. With this filter, frequencies higher than 4.5 kHz can be passed through the filter. The three stages described in the second step of modeling the auditory system can be described as the following relationship^25^:

3 $$y_{an} \left( {t,f} \right) = gh_{c} \left( {\frac{{\partial y_{cochlea} }}{\partial t}\left( {t,f} \right)} \right)*t \mu h_{c} \left( t \right)$$

where ${y}_{an}\left(t,f\right)$ represents the auditory neural pattern obtained through speech signal processing. Next, by applying the Lateral Inhibitory Network (LIN), the discontinuities of the response along the logarithmic frequency for the existing auditory neural pattern should be determined. This LIN can be simulated by the first-order differential in terms of logarithmic frequency and then by using a half-wave rectifier as follows^25^:

4 $$yLIN\left( {t,\;f} \right) = {\text{max}}\left( {\frac{{\partial y_{an} }}{\partial f}\left( {t,\;f} \right),\;0} \right)$$

The last step in the process of modeling the human auditory system is to integrate the result of the above relationship ($yLIN\left(t,f\right)$) in a short range, as follows^25^:

5 $$\mu_{midbrain} \left( {t;\tau } \right) = e^{{ - \frac{t}{\tau }}} u\left( t \right)$$

where u(t) represents the unit step function and $\tau$ defines a short time constant in the range of 2 to 8 ms. With these explanations, the AS, $y\left(t,f\right)$ can be calculated as follows^25^:

6 $$y\left( {t,\;f} \right) = yLIN\left( {t,\;f} \right)*t \mu_{midbrain} \left( {t;\tau } \right)$$

**Figure 2:**
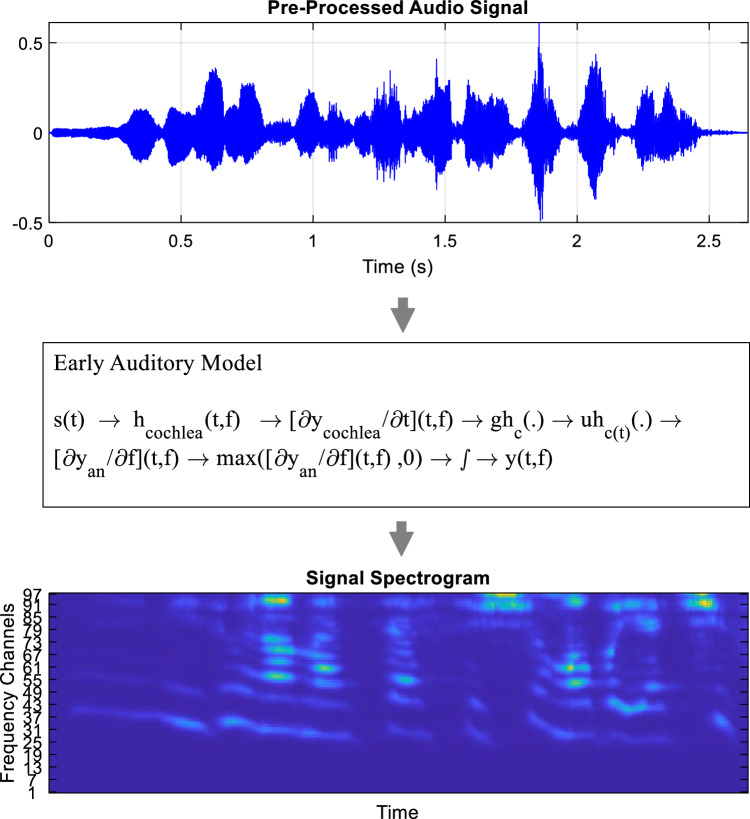
Auditory system modeling diagram.

The described process for modeling the human auditory system is shown as a diagram in Fig. 2. The output matrix resulting from the steps described above is an AS, an example of which is shown in the lower part of Fig. 2.

*Temporal modulation generation based on the AS*: At the higher levels of the human central auditory system, especially in the main cortex of the auditory system, the analysis is performed on the AS by estimating the signal content. In order to model the human auditory system's perception of temporal modulation, in the proposed method, the modulation dimensions analysis is used, in order to provide a more detailed view of the spectro-temporal features of speech signals. The previous works show that by using the logarithmic frequency vector along with the Q discriminator, the best mechanism can be achieved for modeling the human auditory system's perception from temporal modulation^26^. In this way, by applying continuous wavelet transformation to each of the lines in the standard spectrum, the effect of Q can be modeled in an efficient way^27^. In the proposed method, instead of using the standard spectrogram, the AS is used as the input of the modulation dimension analysis step.

The modulation dimension analysis process consists of two main steps. First, given r coefficients, a wavelet filter is applied to each time row in the AS ($y(t,f)$)^25^:

7 $$X^{SP} \left( {r,\;t,\;f} \right) = \frac{1}{r}y\left( {t,\;f} \right)*t {\Psi }\left( { - \frac{t}{r} } \right)$$

By applying (7), the output obtained from each of the cochlear channels can be filtered. In order to reduce complexity and increase computational efficiency, wavelet filters can be simulated by a filter bank, including a set of Gabor filters. Each of these filters can be adjusted for different values of spectro-temporal parameters (low to high speeds and based on Hertz). Modulation rates determined for the set of Gabor filters are $r=\{\mathrm{2,4},\mathrm{8,16,32,64,128,256}\}$.

**Figure 3:**
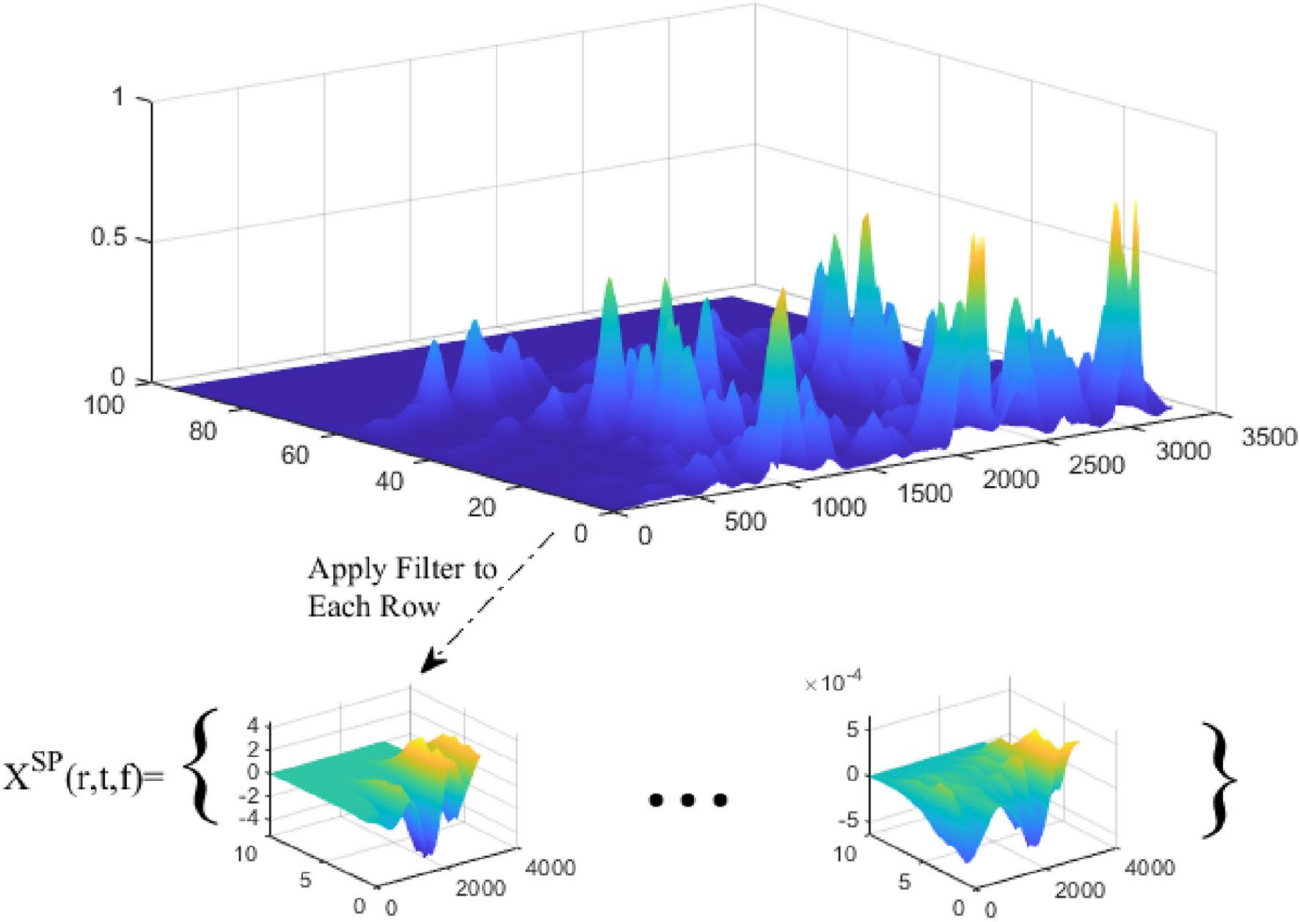
Applying Gabor filters to the row of the spectrogram.

It should be noted that the output obtained in this step uses speed-time–frequency criteria to analyze the input signal. In this way, the obtained AS can be depicted in the form of a three-dimensional spectrogram in terms of speed, time, and frequency. The mentioned filters are applied on each row of this AS. This process is shown in Fig. 3.

In the last step of the temporal modulation generating process based on the AS, by integrating three-dimensional spectrogram with respect to time, the spectro-temporal modulation is achieved.

This process can be implemented as an integration of each member of the set ${X}^{SP}\left(r,t,f\right)$. By doing this, a two-dimensional model is obtained in terms of rate and frequency, and the obtained two-dimensional model is called auditory temporal modulation^25^:

8 $$X^{JF} \left( {r,\;f} \right) = \smallint \left| {X^{SP} \left( {r,\;t,\;f} \right)} \right|^{2} dt$$

**Figure 4:**
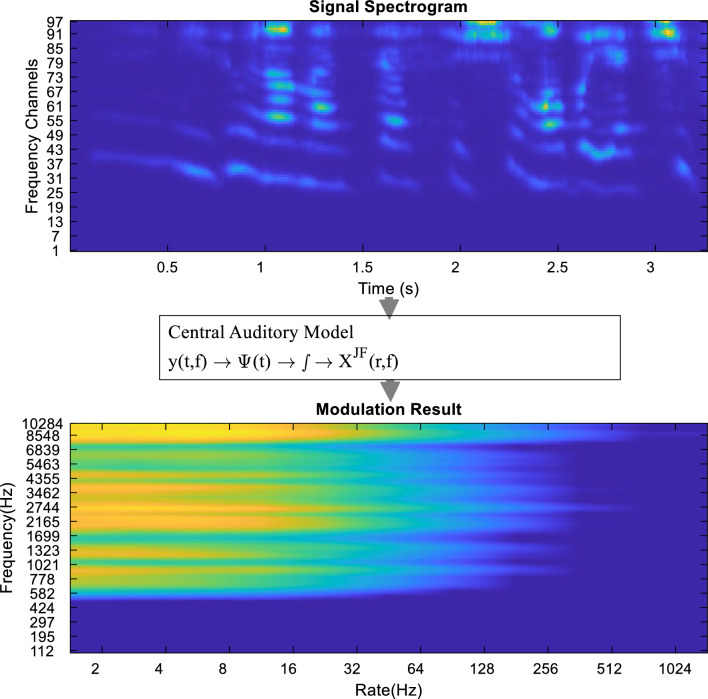
Diagram of the temporal modulation generation steps based on the AS.

The process of generating temporal modulation based on the AS is provided as a diagram in Fig. 4. In short, during this process, Gabor filters are applied on each row of the AS. Then, the integral is taken from the members of the resulting set with respect to time to extract the temporal modulation.

In Fig. 4, the AS is shown in the upper part and the temporal modulation diagram extracted from this AS is shown in the lower part of the figure.

#### Feature extraction with CNN

**Figure 5:**
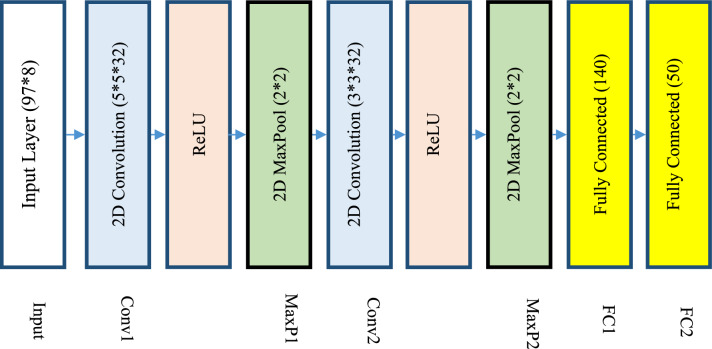
Proposed CNN structure for feature extraction.

In the proposed method, feature extraction is done with a CNN model. It should be noted that this step is only applied to the spectro-temporal modulation features and the entropy features extracted from the speech signal are transferred to the next step without change. The CNN structure used in the proposed method for extracting speech signal features is shown in Fig. 5. In the design of this CNN model, the simplest possible structure for feature extraction is used. After examining different architectures of CNN models for feature extraction, it was determined that the highest performance can be achieved by using a model with two convolution layers, two MaxPool layers, and two fully connected layers.

According to Fig. 5, the proposed CNN model does not have the necessary layers for feature classification, and the output of the last fully connected layer of this model is considered as the extracted features for each sample. The modulation matrices obtained from the previous step have dimensions of 97 × 8, which are applied to the input layer as the input of this CNN model. Two convolution layers consisting of 32 filters with dimensions of 5 and 3 respectively are responsible for extracting input patterns. Finally, the extracted patterns are converted into vectors by using two consecutive fully connected layers and the dimensions of the feature matrix are reduced to 50. The feature vector extracted through this CNN model is combined with entropy features for each sample so that they are finally used as the input of the proposed classification model.

#### Classification based on GC and ECOC

In the last step of the proposed method, the combination of ECOC and GC is used to recognize emotions in speech. The ECOC model is presented as a comprehensive method for solving complex problems, and it is a complete structure for solving multi-class problems through the combination of several binary problems, which can be used to solve problems such as face recognition and emotion recognition, etc. The framework of ECOCs consists of two steps: encryption and decryption. In the coding stage, a code word is assigned to each class in the problem (emotional state). Each code word contains a string of bits, each bit of which indicates the belonging or non-belonging of the class corresponding to that bit for the given binary classification. By combining these codes, a matrix called the code matrix is created, whose rows correspond to the codes created for each of the target classes. In the ECOC model, a binary classifier is assigned to each column of the code matrix, which is trained based on the binary codes corresponding to its column. There are different ways to create the code matrix. In the proposed method, the dense random algorithm^28^ is used to form the code matrix, and $10\times {\mathit{log}}_{2}C$ numbers of binary gamma classification are trained based on this strategy.

After training the GCs based on the columns of the code matrix, the Hamming distance criterion is used to recognize emotional states in new samples. For this purpose, first, the features of each training sample are processed by all GCs so that each classifier creates a binary code as an output. By combining these outputs, a binary string is created, then by matching it with the row of the code matrix, the emotional state in the input sample can be determined. For this purpose, the Hamming distance between the mentioned binary string and each line of the code matrix is calculated, and thus the sample belongs to the class that has the smallest distance. In the following, the mechanism of each GC in the proposed model is explained.

GC is a supervised method whose name is derived from the similarity operator which it uses: the generalized gamma operator. This operator receives two binary vectors *x* and *y* and a positive integer like θ as input and returns 1 if both vectors are the same and 0 otherwise. The gamma operator uses other operators (such as α, β, etc.), which are defined below.

##### Definition 1

 α and β operators: Given the set *A* = {0,1} and *B* = {0,1,3}, the alpha (α) and beta (β) operators are as shown in Table 1.

**Table 1:** $\alpha$ and $\beta$ operators in GC.

$\alpha :A\times A\to B$			$\beta :B\times A\to A$		
x	y	$\alpha (x,y)$	x	y	$\beta (x,y)$
0	0	1	0	0	0
0	1	0	0	1	0
1	0	2	1	0	0
1	1	1	1	1	1
			2	0	1
			2	1	1

##### Definition 2

 α operator: Let $x,y\in {A}^{n}$ be the input column vectors. The output $\alpha (x,y)$ is the *n*-dimensional vector whose components are calculated as follows:

9 $$\alpha \left( {x,\;y} \right)_{i} = \alpha \left( {x_{i} ,\;y_{i} } \right)$$

##### Definition 3

${{\varvec{u}}}_{{\varvec{\beta}}}$ operator: considering the binary pattern *x* ∈ *A*^*n*^ as input, this operator generates the following non-negative integer as output and is calculated as follows:

10 $$u_{\beta } \left( x \right) = \mathop \sum \limits_{i = 1}^{n} \beta \left( {x_{i} ,\;x_{i} } \right)$$

##### Definition 4

 Pruning operator: Let *x* ∈ *A*^*n*^ and *y* ∈ *A*^*m*^ and *n* < *m* be two binary vectors. Then *y* pruned by *x* is displayed as *y*‖*x* and is a binary and n-dimensional vector whose components are calculated as follows:

11 $$\left( {y||_{x} } \right)_{i} = y_{i + m - n} , \left( {i = 1,\;2,\; \ldots ,\;n} \right)$$

The gamma operator requires a binary vector as input. To deal with real vectors or integers, a method to represent these vectors in binary form is needed. In this work, the modified Johnson-Mobius code in^29^ is used. Because the full details of this algorithm have been discussed in ^29^, its process is not explained in this paper.

##### Definition 5

 Gamma operator: Gamma similarity operator takes two binary patterns such as *x* ∈ *A*^*n*^ and *y* ∈ *A*^*m*^ and $n\le m$ and a non-negative integer such as θ as input and generates a binary output for each of the following two states.

##### Case 1

If *n* = *m*, the output is calculated according to the following equation^30^:

12 $$\gamma \left( {x,\;y,\;\theta } \right) = \left\{ {\begin{array}{*{20}l} {1,} \hfill & {if\,m - u_{\beta } \left[ {\alpha \left( {x,\;y} \right)mod\,2} \right] \le \theta } \hfill \\ {0,} \hfill & {otherwise } \hfill \\ \end{array} } \right.$$

In (12), “mod” indicates the remainder of the division.

##### Case 2

If *n* < *m*, the output is calculated using y||_x_ instead of y according to the following equation^30^:

13 $$\gamma \left( {x,\;y,\;\theta } \right) = \left\{ {\begin{array}{*{20}l} {1,} \hfill & {if m - u_{\beta } \left[ {\alpha \left( {x,y||_{x} } \right)mod 2} \right] \le \theta } \hfill \\ {0,} \hfill & {otherwise } \hfill \\ \end{array} } \right.$$

Considering the above operators, the GC operator is explained in the following. Assuming that the basic patterns are $\{\left({x}^{u},{y}^{u}\right)|u=\mathrm{1,2},\dots ,p\}$ with cardinality p and the test pattern is described as $\widetilde{x}\in {R}^{n}$. In this case, the GC classifies the experimental sample x ~ through the following calculation steps:

Step 1 Convert the training base set to binary form using the modified Johnson-Mobius code so that ${e}_{m}$ value is calculated for each component in the n-dimensional vectors of the base set as follows^30^:
  14 $$e_{m} \left( j \right) = \mathop {{\max }^{p}} \limits_{{{\text{i}} = 1}} \left( {x_{j}^{i} } \right), \;\;\forall j \in \left\{ {1,\;2,\; \ldots ,\;n} \right\}$$
Step 2 The stopping parameter is as $\rho ={\underset{\mathrm{j}=1}{\mathrm{max}}}^{n}[{e}_{m}\left(j\right)]$.
Step 3 The test pattern is also modified using the Johnson-Mobius code and coded with the same parameters used to code the original set. If each obtained *e*_*j*_ is greater than its corresponding ${e}_{m}\left(j\right)$, it is coded with more bits.
Step 4 The indicators of all base patterns are converted into two indicators: one for their class and another for their position in the class.
Step 5 The initial value of the parameter $\theta$ is set to zero.
Step 6 If $\theta =0$،, then by calculating $\gamma ({x}_{j}^{u},{\widetilde{x}}_{j},0)$ it is checked whether $\widetilde{x}$ is a fundamental pattern and then the initial weighted addition ${c}_{u}^{0}$ for each basic pattern is calculated as follows^30^:
  15 $$c_{u}^{0} = \mathop \sum \limits_{i = 1}^{n} \gamma \left( {x_{j}^{u} ,\;\tilde{x}_{j} ,\;0} \right),{ }\;\;\;\;{\text{ for u}} = 1,\;2,\; \ldots ,\;{\text{p}}$$
  If there is a unique maximum value equal to n; Then the class corresponding to this maximum value is assigned to the test pattern^30^:
  16 $$\tilde{y} = y^{\sigma } \;\;such\; that\;\; \mathop {{\max }^{p}}\limits_{{{\text{i}} = 1}} c_{i}^{0} = c_{\sigma }^{0} = n$$
Step 7 The value of $\gamma \left({x}_{j}^{i\omega },{\widetilde{x}}_{j},\theta \right)$ is calculated for each component of the basic patterns.
Step 8 The value of the weighted sum c_i_ for each class is calculated^30^ with (17).
  17 $$c_{i} = \frac{{\mathop \sum \nolimits_{\omega = 1}^{{k_{i} }} \mathop \sum \nolimits_{j = 1}^{n} \gamma \left( {x_{j}^{i\omega } ,\;\tilde{x}_{j} ,\;\theta } \right)}}{{k_{i} }}$$
  In the above relationship, k_i_ represents the cardinality of the base set of class $i$.
Step 9 If there is more than one maximum value among different ${c}_{i}$, the value of $\theta$ increases by one unit then steps 7 and 8 are repeated until there is only one maximum value, or the termination condition $\theta >\rho$ is met.
Step 10 If there is a unique maximum value among different c_i_; Then the class corresponding to the experimental pattern $\widetilde{x}$ is calculated as follows^30^:
  18 $$\tilde{y} = y^{j} \;\;such\; that\;\; \max c_{i} = c_{j}$$
Step 11 Otherwise, the pattern $\widetilde{x}$ is assigned to the class with the first maximum value.

## Results

**Figure 6:**
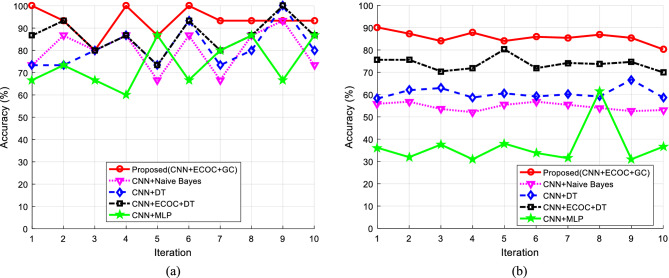
The accuracy of different methods in extracting emotions from two datasets (**a**) Berlin and (**b**) ShEMO during 10 iterations of cross-validation.

The implementation of the proposed method was done to evaluate its performance using MATLAB 2016a software. During these tests, the effectiveness of the proposed method was investigated in terms of accuracy and classification quality, and the results were compared with previous similar works. Also, the speech signals available in ShEMO^22^ and Berlin Emotional^23^ datasets were used as input for experiments. The specification of these datasets were described in Section "Introduction"–"Research method". The evaluation process was done separately for each of these datasets using tenfold cross-validation. Figure 6 shows the results of the accuracy of the proposed method in comparison with other methods for extracting sentiments from the Berlin and ShEMO datasets during 10 iterations of cross-validation. In these graphs, the performance of the proposed method is compared with the case where other learning models use the extracted features of the CNN model.

**Figure 7:**
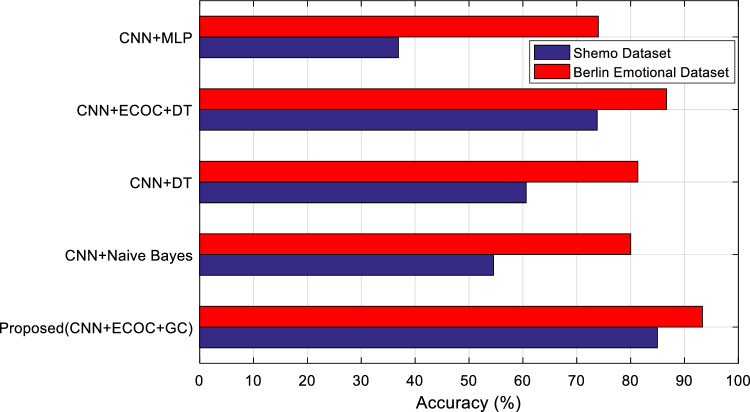
Comparison of the accuracy of different methods for extracting sentiments from the Berlin and ShEMO datasets.

Based on Fig. 6, the proposed method can perform the process of recognizing emotions in speech more accurately in most iterations, and it can be said that it is generally more accurate than the compared methods. Since the only difference between the compared methods is the type of classifier applied to them, and all these methods’ input features are the same; Therefore, the improvement of accuracy obtained in the proposed method can be attributed to the appropriate performance of its classification model. In the proposed method, a combination of ECOC and GC is used in order to recognize emotional states. This combination can correctly recognize emotional states in the Berlin and ShEMO datasets with an average accuracy of 93.33 and 85.73%, respectively. The higher accuracy of the proposed method in the Berlin dataset comes from two factors. First, the data set used from the Berlin dataset includes 5 emotional states. While the number of emotional states in ShEMO is equal to 6 and this increases the complexity of the problem. Secondly, the number of speakers in the ShEMO dataset is much more than the Berlin dataset, which can add to the complexity of the problem. Because each speaker may convey an emotional state through a specific pattern in speech. In Fig. 7, the average accuracy values of the proposed method and other methods are compared for the Berlin and ShEMO datasets.

The Fig. 7 confirms that the proposed method for both Berlin and ShEMO datasets leads to increased recognition accuracy. As mentioned, this improvement can be attributed to the performance of the proposed classification. On the other hand, the combination of ECOC and decision tree has the closest results to the proposed method, which is a direct result of using the ECOC model to overcome the high complexity of multi-class problems.

**Figure 8:**
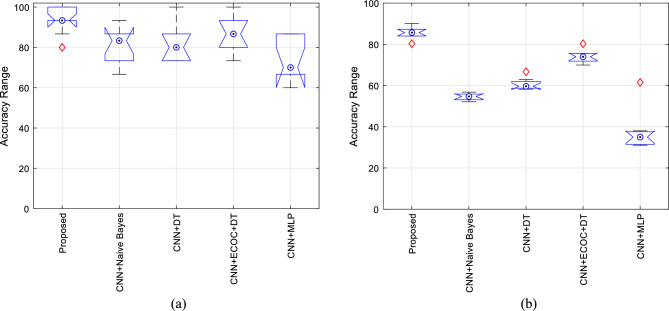
Box plot of different methods accuracy for two datasets (**a**) Berlin and (**b**) ShEMO during 10 iterations of cross-validation.

Inspecting Figs. 6 and 7 shows that the proposed method has two advantages in the recognition process. First, the proposed method can recognize emotions more accurately than other methods. Second, the accuracy variation range of the proposed method is more limited than the compared methods. High accuracy and at the same time, the limitation of its variation range can be considered as a strong point of a recognition system. Because this feature shows the reliability of the outputs generated by that recognition system. These conditions are shown in Fig. 8 for two datasets, Berlin and ShEMO.

In Fig. 8, each box represents the variation range of the accuracy during 10 times of cross-validation. The middle circle indicates the median value of the accuracy during the iterations, and each part of the box shows one of the quartiles of the accuracy changes. Outliers are also drawn as points outside the box. Based on these plots, the proposed method has more compact boxes that are at higher levels than other methods, and this confirms the effectiveness of the proposed method in more accurately recognizing emotions from speech.

**Figure 9:**
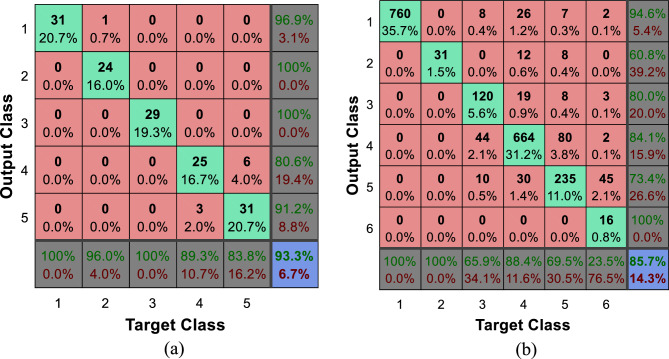
Confusion matrix of the proposed method for recognizing types of emotional states in two datasets (**a**) Berlin and (**b**) ShEMO, after 10 folds of cross-validation.

**Figure 10:**
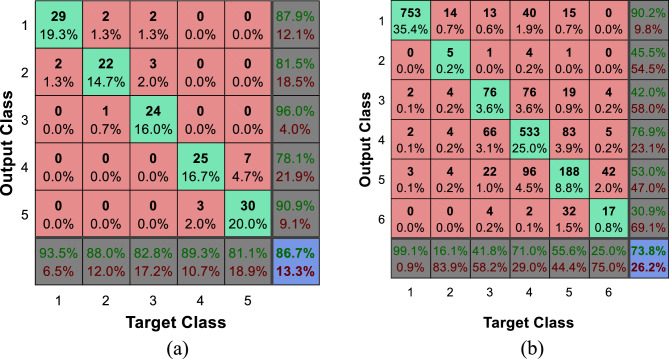
Confusion matrix of the combination of ECOC and decision tree for emotion recognition in the dataset (**a**) Berlin, (**b**) ShEMO, after 10 folds of cross-validation.

The Fig. 9 shows the confusion matrix of the proposed method for recognizing emotional states in the Berlin and ShEMO datasets. The numbering of the classes in these confusion matrices is based on the order of the classes explained at the beginning of this section. For example, the number 2 in the Berlin dataset indicates the state of hatred; While this tag in the ShEMO dataset describes the emotional state of fear. The rows of each confusion matrix represent the outputs of the learning model (recognized emotional states); While the real emotional states of the samples are displayed in the columns of the matrix. In this way, the elements of the main diameter of each matrix represent the number of samples of each emotional state that have been correctly recognized, and the other elements represent the number of errors in classification. Also, the state confusion matrix of emotion recognition by the combination of ECOC and decision tree (the method with the closest performance to the proposed method) is presented in Fig. 10.

Comparison of Figs. 9 and 10 shows that the proposed method can recognize any emotional state with higher accuracy than other methods. By inspecting each emotional state, it can be seen that in the Berlin dataset, the proposed method can recognize samples of each class with higher accuracy. On the other hand, in the ShEMO dataset, the proposed method does not perform well in recognizing samples related to the emotional state of surprise, but it has an acceptable performance in recognizing other emotional states. The relatively small number of surprise class samples can be the main reason for the ineffectiveness of the proposed method in recognizing samples of this class. In addition, the high similarity of the features of this emotional state to the state of sadness in the ShEMO dataset can also be one of the reasons for this; Because about 90% of this class samples are wrongly classified as an emotional state of sadness.

**Figure 11:**
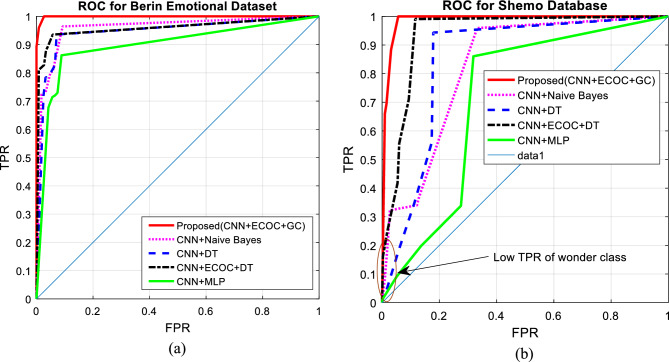
ROC curve for speech emotion recognition in (**a**) Berlin and (**b**) ShEMO datasets after 10 folds of cross-validation.

In Fig. 11, the Received Operating Characteristics (ROC) curve of different methods for recognizing emotional states in the two test bases is presented. In this curve, the values of the true positive rate (TPR) are displayed for changes in the false positive rate (FPR). The goal of any emotion recognition method is to achieve higher TPR values and at the same time, reduce FPR. Therefore, an emotion recognition system with a higher level of the ROC curve has a better performance. Considering that the number of target classes (emotional states) in the research problem is more than two; Therefore, to draw this curve, the values of TPR and FPR were calculated for different classes, and each time, the current emotional state was considered as a positive class and other classes as a negative class.

The graphs depicted in Fig. 11, show that the proposed method can achieve higher TPR and lower FPR values for both tested datasets. These results confirm that the proposed method is superior in recognizing emotional states separately for each class. The ROC curve in Fig. 11b for the ShEMO dataset shows that the proposed method has a lower TPR at the initial points of the curve than the combination of ECOC and decision tree. This low TPR results from the inappropriate performance of the proposed method in recognizing the emotional state of surprise, which was discussed in Fig. 9. However, the proposed method has worked well in accurately recognizing other emotional states, which results in achieving higher levels of the ROC curve.

To better check the effectiveness of the proposed method, the precision, recall, and F-Measure criteria can be used. The precision criterion, can demonstrate the accuracy of system in recognizing samples of each target class, separately. Also, the recall criterion shows that which ratio of samples belonging to each target class has been recognized correctly. Finally, the F-Measure is used to describe the classification efficiency of the system by harmonic mean of precision and recall. These criteria are formulated by Eqs. (19) to (21):

19 $$Precision = \frac{TP}{{TP + FP}}$$

20 $$Recall = \frac{TP}{{TP + FN}}$$

21 $$F - Measure = 2 \times \frac{Precision \times Recall}{{Precision + Recall}}$$

Since these criteria consider the classification task as a binary problem, they are calculated for each target class, separately. Therefore, for calculating these criteria for each target class, the mentioned class is considered as positive while the other classes are assumed to be negative. In the above equations, TP refers to the number of correctly recognized samples belonging to positive class. Also, FP demonstrates the number of samples which actually belong to negative class but are classified as positive. Finally, FN refers to the number of positive samples which have been incorrectly labeled as positive.

**Figure 12:**
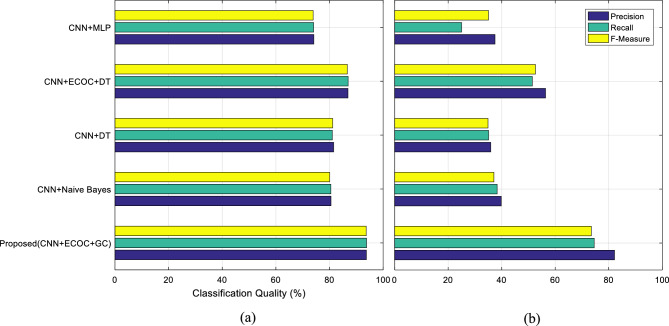
Classification rates of speech emotion recognition in (**a**) Berlin and (**b**) ShEMO datasets.

In Fig. 12, the efficiency of the proposed method is compared with other methods in terms of precision, recall, and F-Measure criteria. Also, the numerical results related to these criteria are given in Table 2.

**Table 2:** Comparing the efficiency of the proposed method with other classification models.

Dataset	Method	Accuracy	F-measure	Recall	Precision
Berlin	Proposed	93.3333	93.6883	93.8139	93.7393
	NB^31^	80.0000	80.0573	80.4921	80.5399
	DT^32^	81.3333	81.1562	81.0514	81.5426
	ECOC + DT^33^	86.6667	86.6354	86.9348	86.8789
	MLP^34^	74.0000	73.8902	74.0701	74.1400
	Kerkeni et al.^10^	82.4100	82.1761	83.0122	81.3567
ShEMO	Proposed	85.7277	73.4749	74.5676	82.1529
	NB^31^	54.5540	37.0834	38.3228	39.8235
	DT^32^	60.6103	34.8818	35.1087	35.9131
	ECOC + DT^33^	73.8028	52.6375	51.4266	56.4003
	MLP^34^	36.9014	35.0632	25.0046	37.5138
	Liou et al.^16^	70.5333	69.6606	71.0404	68.3333

Comparing precision, recall, and F-Measure values in Fig. 12 and Table 2 shows that the proposed method can be more successful in separating the emotional states of each class. These results confirm the claim made in this article regarding the greater effectiveness of the combination of ECOC and GC for more accurate recognition of emotions in speech. On the other hand, the comparison of the criteria values in Table 2 also confirms that this model can classify emotional states more accurately than previous methods.

The research conducted in^14^ used the ShEMO dataset to recognize emotional states, but this method was limited to recognizing only two emotional states: anger and neutral. Therefore, in order to compare the effectiveness of the proposed method with the results of this research, other target classes are ignored and emotion recognition is done only based on samples belonging to the two classes "anger" and "neutral".

The method in^14^ can perform two emotional states "anger" and "neutral" without separating the gender samples with 90.97% accuracy. Meanwhile, by using the proposed method, these two emotional states can be separated with an accuracy of 93.23%.

**Figure 13:**
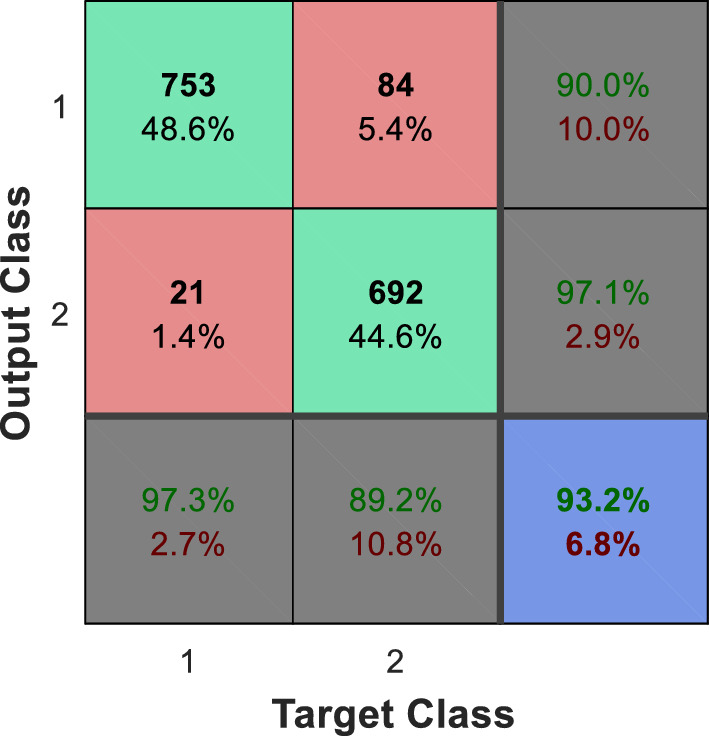
Confusion matrix of the proposed method for recognizing two emotional states of anger and neutral.

**Figure 14:**
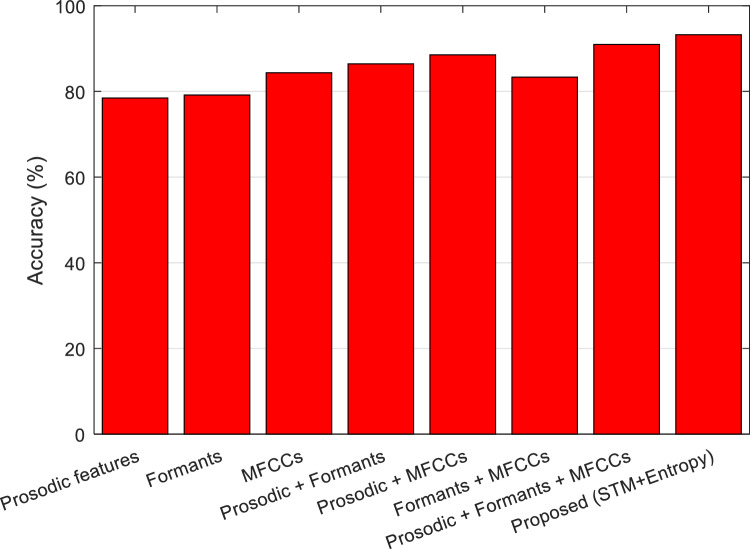
Accuracy comparison of the proposed method with other feature extraction methods for recognizing the two emotional states of anger and neutral.

These results show that by using the proposed method, the recognition accuracy can be increased by 2.26% compared to the method of^14^. Figure 13 shows the confusion matrix resulting from the recognition of the two mentioned emotional states by the proposed method. Also, in Fig. 14, the accuracy of the proposed method is compared with other speech feature extraction methods.

These results clearly show that by using the combination of spectro-temporal modulation and entropy features in the proposed method, the accuracy of recognizing emotional states can be increased compared to previous methods.

## Discussion

Using the experiments conducted in this research, it was attempted to identify the advantages and limitations of the proposed strategy from different aspects. In this regard, two different datasets that include various emotions were used to conduct experiments in order to prove the generality of the proposed strategy. The evaluation and comparison of the proposed method in terms of identification accuracy (Figs. 6 and 7) and its variation range (Fig. 8) showed that the combination of ECOC and GC can perform better than the compared classifications (such as the combination of ECOC with NB or DT), and achieved higher accuracy values in both tested data sets. In addition, the range of accuracy changes reported by the proposed method during CV folds has been narrower than other methods, which confirms the higher reliability of the output of this model. Nevertheless, the examination of confusion matrices (Figs. 9 and 10) for two datasets, ShEMO and Berlin, showed that the proposed method is more successful in recognizing each emotion than other classifiers. Examining the ROC curve (Fig. 11) and the classification rates (Fig. 12) proved this claim. The higher precision values show that this model is more accurate in assigning labels of each target category to samples, and the superiority of the recall criterion confirms the higher efficiency of the proposed method in recognizing samples of each emotional state.

Overall, these results showed that the proposed method performs better than methods such as Kerkeni et al.^10^ and Liou et al.^16^ for both Berlin and ShEMO datasets. This superiority can be attributed to two main factors: First, the combination of ECOC and GC has led to the formation of a more powerful classification model for identifying emotional states, which can be used to resist the increased complexity caused by the increase in the number of emotional states. Secondly, the use of STM has made it possible to represent emotional states in speech signals in a more efficient way, and the extraction of STM features by CNN and the combination of entropy features with it can be effective in increasing the accuracy of the model by at least 2.26%. The results presented in Figs. 13 and 14 prove this claim. Based on these results, the combination of STM and entropy features leads to higher accuracy compared to conventional feature description models such as MFCC, Formants or Prosodic^14^.

One of the limitations of the proposed method is the need for higher computing power of its learning model, which results from the use of several GCs in the ECOC model. This has caused the processing time of the proposed method to train the learning model to be more than the conventional methods. Although this increase in processing time only occurs in the training phase; this problem can be solved by using parallel processing techniques. In future works, the use of other feature extraction techniques to describe the emotional features of speech can be investigated. Also, combining the ECOC model with other existing classifiers to achieve a more accurate emotion recognition system can be a topic for further research.

## Conclusion

In this paper, a new method is presented to recognize emotion in speech using machine learning techniques. The proposed method uses a set of entropy features and spectro-temporal modulation to describe speech features, and the feature extraction is done by a CNN. Also, a new model based on the combination of GC and ECOC is utilized to classify features and recognize emotional states. These two techniques make it possible to recognize the emotional states of speech with higher accuracy and efficiency in comparison with previous works. The performance of the proposed method was tested through two datasets, Berlin and ShEMO, and the results were compared with previous similar works. The obtained results showed that the proposed method is an accurate solution in recognizing the emotional states of speech and could recognize speech emotions in the Berlin and ShEMO datasets with an average accuracy of 93.33 and 85.73%, respectively, which had an improvement of at least 2.1% compared to the compared methods. On the other hand, comparing the performance of feature extraction techniques in recognizing emotional states showed that by combining spectro-temporal modulation and entropy in the proposed method, the accuracy of recognizing emotional states can be 2.26% higher than compared methods.

## Data Availability

All data generated or analyzed during this study are included in this published article.
